# Combining Hepatic and Splenic CT Radiomic Features Improves Radiomic Analysis Performance for Liver Fibrosis Staging

**DOI:** 10.3390/diagnostics12020550

**Published:** 2022-02-21

**Authors:** Yunchao Yin, Derya Yakar, Rudi A. J. O. Dierckx, Kim B. Mouridsen, Thomas C. Kwee, Robbert J. de Haas

**Affiliations:** 1 Department of Radiology, Medical Imaging Center Groningen, University of Groningen, University Medical Center Groningen, P.O. Box 30001, 9700 RB Groningen, The Netherlands; y.yin@umcg.nl (Y.Y.); d.yakar@umcg.nl (D.Y.); r.a.dierckx@umcg.nl (R.A.J.O.D.); kim@cercare-medical.com (K.B.M.); t.c.kwee@umcg.nl (T.C.K.); 2 Department of Clinical Medicine, Center of Functionally Integrative Neuroscience, Aarhus University, 8000 Aarhus, Denmark

**Keywords:** liver, artificial intelligence, machine learning, radiomics, multidetector computed tomography

## Abstract

***Background:*** The exact focus of computed tomography (CT)-based artificial intelligence techniques when staging liver fibrosis is still not exactly known. This study aimed to determine both the added value of splenic information to hepatic information, and the correlation between important radiomic features and information exploited by deep learning models for liver fibrosis staging by CT-based radiomics. ***Methods*:** The study design is retrospective. Radiomic features were extracted from both liver and spleen on portal venous phase CT images of 252 consecutive patients with histologically proven liver fibrosis stages between 2006 and 2018. The radiomics analyses for liver fibrosis staging were done by hepatic and hepatic–splenic features, respectively. The most predictive radiomic features were automatically selected by machine learning models. ***Results***: When using splenic–hepatic features in the CT-based radiomics analysis, the average accuracy rates for significant fibrosis, advanced fibrosis, and cirrhosis were 88%, 82%, and 86%, and area under the receiver operating characteristic curves (AUCs) were 0.92, 0.81, and 0.85. The AUC of hepatic–splenic-based radiomics analysis with the ensemble classifier was 7% larger than that of hepatic-based analysis (*p* < 0.05). The most important features selected by machine learning models included both hepatic and splenic features, and they were consistent with the location maps indicating the focus of deep learning when predicting liver fibrosis stage. ***Conclusions:*** Adding CT-based splenic radiomic features to hepatic radiomic features increases radiomics analysis performance for liver fibrosis staging. The most important features of the radiomics analysis were consistent with the information exploited by deep learning.

## 1. Introduction

Chronic liver disease prevalence is increasing globally [1]. Repetitive liver damage, secondary to any cause of liver injury, results in progressive fibrosis, disrupted hepatic architecture, and aberrant regeneration [2]. Cirrhosis is considered end-stage liver disease and puts patients at risk for the development of hepatocellular carcinoma, portal hypertension, and liver failure. Currently, liver transplantation can be a lifesaving procedure for cirrhotic patients; however, demand greatly outweighs donor organ supply [3]. To allow early intervention to avoid or delay clinical decompensation and the need for liver transplantation, early diagnosis and adequate staging of liver fibrosis are of utmost importance [4].

Histopathology is currently considered the gold standard for liver fibrosis diagnosis and staging. To enable histopathological examination of liver parenchyma, a percutaneous image-guided biopsy can be performed. However, up to 6% of patients experience postprocedural complications, with a reported mortality rate of 1.6% [5].

To overcome these disadvantages, various radiology-based liver fibrosis staging methods have been explored to replace biopsy, with among them, radiomics analysis [6,7,8] and deep learning techniques [9,10,11]. In a recent study, a convolutional neural network (CNN) was used to stage liver fibrosis, and location maps were generated by gradient-weighted class activation mapping (Grad-cam) [12], which indicated the focus of the CNN when predicting liver fibrosis stage [13]. These location maps illustrated that the CNN used not only information from the liver but also the spleen. Compared with deep learning, radiomics analysis uses manually designed features extracted from computed tomography (CT) scans instead of the raw image and requires less data and computational power for training. Furthermore, the model of radiomics analysis can indicate which kind of symptoms on images are more important for the model by giving radiomic features importance. Until now, radiomics analysis for liver fibrosis staging based on both hepatic and splenic features has not yet been performed. Whether adding splenic features to hepatic features could improve the radiomics analysis performance for liver fibrosis staging on CT images, and whether radiomic analysis and deep learning use similar information from CT images to predict liver fibrosis stages, is unclear.

Therefore, the primary aim of the current study was to determine whether adding splenic information to hepatic information can improve the performance of liver fibrosis staging by CT-based radiomics analysis and to identify the most important radiomic features in this setting. The secondary aim was to explore the correlation between important radiomic features and location maps generated by deep learning models.

## 2. Materials and Methods

### 2.1. Study Population

**Figure 1:**
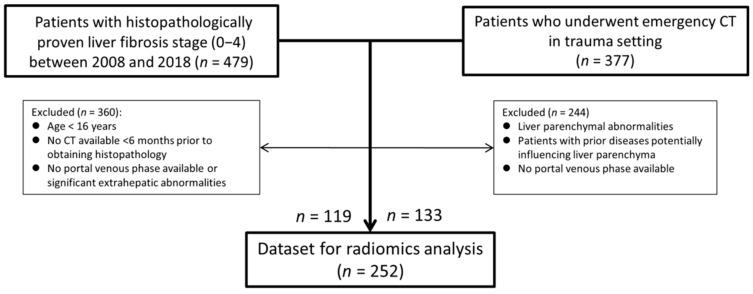
Flowchart of the study population. Abbreviation: CT = computed tomography.

All consecutive patients who were diagnosed with liver fibrosis at our tertiary referral center between 2006 and 2018, aged >16 years, and of whom both a contrast-enhanced portal venous phase CT scan of the upper abdomen (image acquisition parameters: automatic tube current modulation and tube voltage selection; slice thickness 1 mm; pitch 0.6) and histopathological proof of liver fibrosis stage were available, with less than 6 months between them, were included in the study (Figure 1). Patients with focal liver lesions were excluded from the study.

To improve the balance between patients with and without fibrosis (in whom generally no histopathological examination of liver tissue is performed), patients who underwent a contrast-enhanced abdominal CT scan in the portal venous phase (similar image acquisition parameters as mentioned earlier) at the emergency department to rule out traumatic injuries during the same inclusion period were added to our study population. Patients were only included if no focal liver lesions, injuries, or other abnormalities of liver morphology were present, as well as no history of liver disease or associated risk factors.

Patients were identified in our institutional database and retrospectively analyzed. The study was approved by the local institutional review board, and the need for obtaining informed consent was waived.

### 2.2. Reference Standard

The reference standard consisted of histopathological examination of liver parenchyma, either through liver biopsy, liver resection, or after liver transplantation. Liver fibrosis stage was determined by a specialized liver pathologist according to the METAVIR staging system [14]. This system consists of five stages including no signs of liver fibrosis (F0), fibrosis of portal area without septa (F1), portal fibrosis with few septa (F2), septal fibrosis without cirrhosis (F3), and cirrhosis (F4).

### 2.3. Image Processing and Feature Extraction for Radiomics Analysis

**Figure 2:**
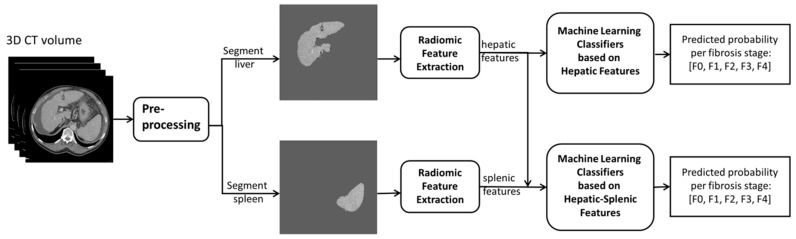
Overall algorithm scheme. First, the computed tomography (CT) volume was preprocessed; then, the liver and spleen were segmented as the region of interest. The radiomic features were extracted from the segmented liver and spleen. The machine learning classifiers were trained by hepatic features and hepatic–splenic features, respectively, to predict the probability array of liver fibrosis stages, namely F0–F4.

The algorithm scheme is shown in Figure 2. The CT scans were first resampled to the same thickness of 2.0 mm. A soft tissue window (Width: 400 Hounsfield Units, Level: 50 Hounsfield Units) was applied on the resampled CT image. The liver and spleen parenchyma were first segmented by V-nets [15] pretrained by public datasets IRCADB and Medical Segmentation Decathlon, respectively. The segmentation was checked for correctness by a research fellow (Y.Y.) under the supervision of an abdominal radiologist (R.J.d.H.; 8 years dedicated experience) and served as the region of interest used for the radiomics analysis.

The radiomic features were then extracted from the original 3D images of the segmented liver and spleen parenchyma, respectively. The images were resampled with multiple spacing parameters by sitkBSpline interpolator provided by the pyradiomics library [16], including [1.0 mm, 1.0 mm, 1.0 mm], [1.0 mm, 1.0 mm, 2.0 mm], and [1.0 mm, 1.0 mm, 5.0 mm]. The parameters [1.0 mm, 1.0 mm, 2.0 mm] were used as resampled spacing for the following experiments because of the machine learning model performance. The images were quantified by multiple fixed bin widths of 1, 10, and 25, and the bin size of 10 was chosen for the subsequent experiments. We extracted 107 features from different categories according to the Image Biomarker Standardization Initiative, including 18 first-order statistics, 14 3D shape-based features, 24 gray level co-occurrence matrix features, 16 gray level size zone matrix features, 16 gray level run length matrix features, 5 neighboring gray tone difference matrix features, and 14 gray level dependence matrix features [17]. The feature extraction was executed using the open-source library Pyradiomics 3.0 [16].

To remove the features that did not show differences among liver fibrosis stages, we performed univariate feature selection by the Kruskal–Wallis test. Features statistically related to the liver fibrosis stage (*p* < 0.05) were included in the selected feature subset.

### 2.4. Radiomics Analysis for Liver Fibrosis Staging

Machine learning classifiers were trained by standardized hepatic features and hepatic–splenic features with zero mean and unit variance. The hepatic features-based classifier and hepatic–splenic feature-based classifier were tested by CT scans from the same group of patients and their performance was compared. To avoid bias toward machine learning model selection, a variety of commonly used classifiers was chosen in the experiments, including multinomial logistic regression with L1 and L2 penalty and tree-based ensemble classifiers based on AdaBoosting [18], Gradient Boosting [19], and XGBoosting [20] methods.

To better understand the prediction made by machine learning classifiers, we evaluated the top 5 most important features of the trained classifiers. For logistic regression, the importance was decided by the weights of radiomic features, which represent the feature’s relevance to fibrosis stage F0 and stage F4. For ensemble classifiers, the importance was decided by Gini importance [21], which is commonly used to evaluate features in boosting methods. More detailed information can be found in Appendix A.

### 2.5. Evaluation and Statistical Analysis

The liver fibrosis staging classifiers were trained by the fivefold cross-validation method and tested on 20% of the study population. To make the radiomics analysis results comparable with recently reported artificial-intelligence-based liver fibrosis staging projects [9,10,11,13], we grouped liver fibrosis stages into significant fibrosis (F2−4), advanced fibrosis (F3−4), and cirrhosis (F4). The area under the receiver operating characteristic (ROC) curves (AUC) and the accuracies of discriminating significant fibrosis (F0−1 vs. F2−4), advanced fibrosis (F0−2 vs. F3−4), and cirrhosis (F0−3 vs. F4) were used to determine the performance of liver fibrosis staging classifiers on each class. The accuracy and micro-average AUC were used to evaluate the liver fibrosis staging classifiers’ performance as a multistage classification.

Furthermore, the microaveraged AUCs of classifiers based on hepatic–splenic features and merely hepatic features were compared utilizing bootstrapping of 1000 repetitions; statistical significance was set at *p* ≤ 0.05. The analysis was performed by Y.Y. with Python 3.7.9 and library scikit-learn 0.23.2 [22].

## 3. Results

### 3.1. Study Population

In total, 252 patients were included in the radiomics analysis for liver fibrosis staging (Figure 1). Patient demographics are summarized in Table 1. The median age of the patients was 59 years (interquartile range: 48−65 years), and 140 patients (55.6%) were men. One hundred thirty-four patients (53.2%) had no signs of liver fibrosis (F0), 8 patients (3.2%) had fibrosis stage F1, 10 patients (4.0%) had fibrosis stage F2, 18 patients (7.1%) had fibrosis stage F3, and 82 patients (32.5%) had cirrhosis (F4). Two patients who underwent a liver transplantation were included twice in the dataset, as the histopathology results were based on different livers (i.e., the native liver before transplantation and the transplanted liver after retransplantation).

### 3.2. Radiomic Features

**Figure 3:**
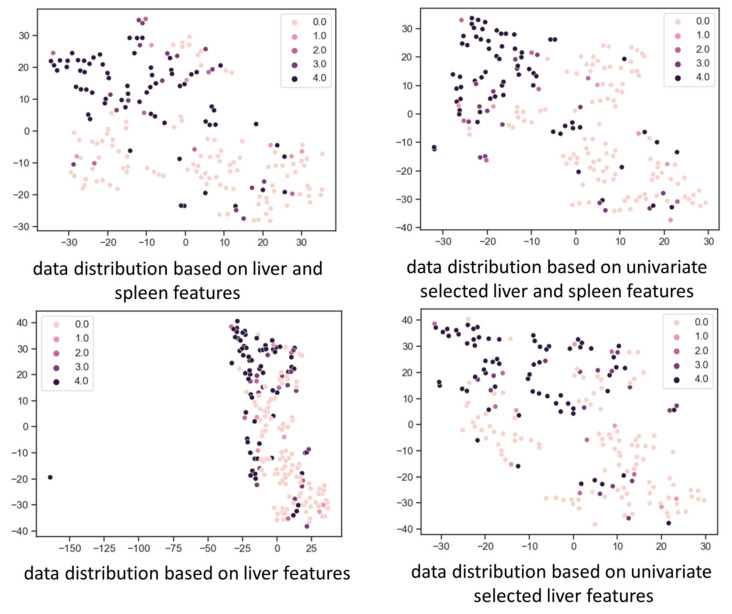
Data distribution based on radiomic features after dimension reduction using the *t*-distributed stochastic neighbor embedding (*t*-SNE) method. Features are reduced to two dimensions represented by the two axes. Each dot represents a computed tomography (CT) scan, and its color represents the liver fibrosis stage as shown at the upper corner of the graph. A relative clustering of different fibrosis stages is observed, which means that CT scans of different fibrosis stages can be differentiated based on the two-dimensional features after dimension reduction.

After extracting the radiomic features from both the liver and spleen, 77 features from the liver and 65 features from the spleen were selected by the univariate feature selection (*p <* 0.05). To visualize data distribution, principal component analysis and *t*-distributed stochastic neighbor embedding (*t*-SNE) were combined to reduce the radiomic features to two dimensions, represented by the axes in the plot of Figure 3. According to the data visualization, a relatively clear clustering of F0 and F4 liver fibrosis stages can be observed, indicating that CT scans of both groups can be differentiated based on two-dimensional features after dimensionality reduction, which supported the utility of the following experiments.

### 3.3. Performance of Liver Fibrosis Stage Classifiers

Table 2 shows the performance of logistic regression classifiers and tree-based ensemble classifiers evaluated by the accuracy and micro-averaged AUC. The AUC and accuracy of significant fibrosis (F0−1 vs. F2−4), advanced fibrosis (F0−2 vs. F3−4), and cirrhosis (F0−3 vs. F4) can also be found in Table 2. The accuracy of L1 regulated logistic regression classifiers increased by 8% after adding splenic features to hepatic features. Compared with logistic regression classifiers, the AUC of ensemble classifiers improved more significantly when adding splenic features. Especially for the XGBoosting classifier, the microaveraged AUC increased by 7%. Bootstrapping with 1000 repetitions was used to compare the results between hepatic and hepatic–splenic-based classifiers [23]. The microaveraged AUCs of AdaBoosting, Gradient Boosting, and XGBoosting classifiers based on hepatic–splenic features were significantly larger than when based on hepatic features alone (*p* = 0.025, *p* = 0.024, and *p* < 0.001, respectively), while the microaveraged AUCs of logistic regression were not (*p* = 0.243 and *p* = 0.277). In addition, we used full feature sets without univariate feature selection, which showed a similar result; the AUCs of hepatic–splenic classifiers improved by 5% at most.

### 3.4. Top 5 Weighted Features of the Trained Classifiers

The top 5 weighted features of logistic regression classifiers are shown in Table 3. Overall, one first order, three shape-based, and five texture-based liver features, as well as four shape-based and one texture-based splenic features were among the top five weighted features used by the logistic regression classifiers to stage liver fibrosis. The shape-based features concern the smallest axis length of liver-enclosing ellipsoid, the flatness of 3D liver, the second-largest axis length of spleen-enclosing ellipsoid, the surface area of the spleen, and the largest diameter in sagittal direction of both liver and spleen. The texture-based features include texture heterogeneity, coarseness, variability of gray-level intensity, the concentration of low gray-level values, the deviation of gray-level intensity to the mean gray-level in liver tissue, and the asymmetry of the gray value distribution in the splenic tissue compared with the mean value. Of note, all analyzed top five important features positively related to F0 liver fibrosis are only liver features and 60% are shape-based hepatic features; however, the features positively related to F4 liver fibrosis (i.e., cirrhosis) concern both liver and splenic features.

The top five weighted features of tree-based ensemble classifiers for liver fibrosis staging are shown in Table 4. Overall, one first order, three shape-based, and four texture-based liver features, and one first order and four texture-based splenic features were among the five most important features for ensemble classifiers. The shape-based features include liver volume, the flatness of the 3D liver, and the largest liver diameter in the sagittal direction. The liver texture features concern the variability and variance of size zone volume with similar gray-level, the joint distribution of larger size zones with higher gray-level values, the similarity of run lengths throughout liver tissue, and the visibility of primitives in liver tissue. The splenic texture features reflect the concentration of low gray-level, texture complexity, variance in gray-level runs, and joint distribution of larger size zones with higher gray-level values.

## 4. Discussion

This study aimed to determine the value of adding splenic information to liver information in liver fibrosis staging by CT-based radiomics analysis, as well as investigate the correlation between important features of radiomic analysis and the CT-based information exploited by the deep learning model for liver fibrosis staging.

In the current study, the microaveraged AUC of best-performing radiomics analysis based on hepatic–splenic features was significantly larger than based on merely hepatic features, namely the XGBoosting classifier (*p* < 0.01). Interestingly, the logistic regression classifiers demonstrated that hepatic radiomic features, especially shape-based hepatic features that can be extracted from the liver surface, were most important for liver fibrosis stage F0. The combination of hepatic and splenic texture features was much more important when it concerned liver fibrosis stage F4. This is in accordance with a recent study in which a deep learning model was trained for liver fibrosis staging and location maps were generated by Grad-cam [12] to highlight the region on the CT scan that the model exploited information from [13]. More specifically, the location maps indicated the deep learning model focused more on the surface of the liver when giving the prediction of fibrosis stage F0, while more attention was paid on the liver parenchyma and spleen when giving the prediction of fibrosis stage F4 [13]. The information given by these location maps was consistent with the most important features chosen by machine learning in the current study.

From a clinical point of view, it seems logical that radiomic analysis and deep learning models use both hepatic and splenic radiomic information to stage liver fibrosis. The liver develops morphological changes when liver fibrosis progresses, meanwhile the spleen also often increases in size and parenchymal changes can occur such as the development of splenic siderotic nodules.

In a recent study by Nitsch et al., magnetic resonance imaging (MRI)-based radiomic features and random forest classifier were used to predict the severity of liver cirrhosis, with clinical decompensation and model for end-stage liver disease (MELD) scores as reference standard. Nitsch et al. concluded that adding splenic MRI-based radiomic features can increase the AUC for predicting a higher median MELD score or clinical decompensation [24]. However, their study lacked an objective reference standard and was only focused on patients with known cirrhosis. In our study, histopathology was used as reference standard, which is still considered the most objective staging method of liver fibrosis. In addition, our study not only focused on F4 liver fibrosis (i.e., cirrhosis), but also other liver fibrosis stages were included, thereby offering the opportunity to determine differences in types of radiomic features used for different liver fibrosis stages. In this way, the added value of introducing splenic radiomic features becomes even more clear.

Our study found that the machine learning models based on splenic–hepatic features could outperform the models only based on hepatic features. To generalize further, it might be helpful to include information of multiple organs when developing computer-aided diagnostic tools for systemic diseases such as liver fibrosis. In addition, as mentioned earlier, the most important radiomic features selected by machine learning models in the current study were consistent with the location maps generated by a recently reported liver fibrosis staging network [13]. The results of the current radiomic analysis could further support the location maps generated by Grad-cam, showing the focus of deep learning models when predicting liver fibrosis stages. This might lead to wider acceptance of artificial intelligence techniques in daily clinical practice. More specifically for liver fibrosis patients, our results might be the first step toward increased acceptance of artificial intelligence techniques as part of the decision-making process in the treatment of these patients, thereby ensuring early intervention and perhaps reducing the need for liver transplantation.

Some limitations of our study have to be mentioned. First, our study is a retrospective and single-center study, which might limit the utility of our results in broader clinical practices. A prospective and multicenter study using the same research question could be a possible improvement in the future. Second, the relatively small number of patients with F1, F2, and F3 liver fibrosis in the current study can be considered a limitation. This renders the performance of machine learning models sensitive to the bias-variance tradeoff. Unfortunately, because histopathological proof in patients with less severe liver fibrosis stages is generally not available, it is difficult to prevent imbalances in the dataset. In addition, the stability of texture features could be influenced by the parameters used during CT image acquisition and reconstruction techniques could also influence the robustness of radiomic features [25,26], which might further influence the radiomic analysis. These factors should also be subject of future research.

## 5. Conclusions

Adding CT-based splenic radiomic features to hepatic radiomic features increases radiomic analysis performance for liver fibrosis staging. In addition, the most important features of the radiomic analysis were consistent with location maps generated by deep learning, which could improve physicians’ confidence when using deep learning techniques more regularly in daily practice.

## Figures and Tables

**Table 1:** Demographics of the study population.

Variable		Total Cohort	Liver Fibrosis Stage				
			F0	F1	F2	F3	F4
Total number of patients		252	134	8	10	18	82
Median age (interquartile range)		59 (48−65)	63 (50-−74)	64 (38−71)	57 (43−64)	48 (43−62)	60 (54−65)
Gender	Male	140 (55.6%)	68 (50.7%)	3 (37.5%)	7 (70.0%)	11 (61.1%)	51 (62.2%)
	Female	112 (44.4%)	66 (49.3%)	5 (62.5%)	3 (30.0%)	7 (38.9%)	31 (37.8%)
Etiology of liver fibrosis	Alcoholic	26 (22.0%)	-	0	0	1	25
	Autoimmune hepatitis	5 (4.2%)	-	0	1	1	3
	HBV	3 (2.5%)	-	0	0	0	3
	HCV	10 (8.5%)	-	0	0	1	9
	PSC	3 (2.5%)	-	0	2	1	0
	Steato- hepatitis	8 (6.8%)	-	0	0	0	8
	Wilson disease	1 (0.8%)	-	0	0	0	1
	Other	17 (14.4%)	-	1	0	7	9
	Unknown	45 (38.1)	-	7	7	7	24

Abbreviations: HBV = hepatitis B virus; HCV = hepatitis C virus; PSC = primary sclerosing cholangitis.

**Table 2:** Performance of machine learning classifiers for liver fibrosis staging.

Machine Learning Classifier Type	Training Features	Accuracy (%; 95% CI)	Microaveraged AUC (95% CI)	AUC (95% CI)			Accuracy (%; 95% CI)		
				Significant Fibrosis	Advanced Fibrosis	Cirrhosis	Significant Fibrosis	Advanced Fibrosis	Cirrhosis
Logistic regression classifier with L1 penalty	Selected liver features	76 (70,83)	0.94 (0.93,0.97)	0.95 (0.93,0.98)	0.92 (0.88,0.95)	0.91 (0.86,0.95)	86 (78,90)	80 (75,88)	82 (78,100)
	Selected liver & spleen features	84 (80,90)	0.95 * (0.93,0.98)	0.93 (0.90,0.98)	0.88 (0.84,0.94)	0.94 (0.90,0.99)	92 (83,95)	86 (83,95)	90 (88,100)
Logistic regression classifier with L2 penalty	Selected liver features	78 (73,85)	0.95 (0.93,0.97)	0.96 (0.95,0.99)	0.91 (0.88,0.95)	0.93 (0.90,0.97)	88 (78,93)	82 (78,88)	84 (80,100)
	Selected liver & spleen features	80 (75,85)	0.95 ** (0.93,0.97)	0.95 (0.93,0.98)	0.90 (0.86,0.95)	0.94 (0.91,0.97)	88 (78,93)	81 (78,90)	86 (83,100)
AdaBoosting	Selected liver features	74 (68,80)	0.82 (0.79,0.86)	0.74 (0.67,0.80)	0.72 (0.63,0.79)	0.82 (0.73,0.88)	76 (70,84)	80 (75,100)	74 (67,80)
	Selected liver & spleen features	76 (70,83)	0.84 *** (0.82,0.88)	0.57 (0.48,0.66)	0.61 (0.52,0.69)	0.84 (0.75,0.90)	78 (73,85)	82 (78,100)	57 (48,66)
Gradient Boosting	Selected liver features	76 (70,83)	0.88 (0.85,0.93)	0.85 (0.81,0.92)	0.83 (0.78,0.89)	0.88 (0.78,0.93)	80 (75,85)	80 (75,100)	85 (81,92)
	Selected liver & spleen features	78 (73,85)	0.91 **** (0.88,0.95)	0.84 (0.80,0.90)	0.88 (0.85,0.94)	0.86 (0.78,0.90)	80 (75,88)	84 (80,100)	84 (80,90)
XGBoosting	Selected liver features	80 (75,88)	0.86 (0.82,0.91)	0.78 (0.70,0.87)	0.79 (0.71,0.89)	0.90 (0.83,0.95)	84 (80,90)	86 (83,100)	78 (70,87)
	Selected liver & spleen features	82 (78,88)	0.93 ***** (0.90,0.96)	0.86 (0.82,0.92)	0.90 (0.86,0.94)	0.90 (0.80,0.95)	84 (80,93)	88 (85,100)	86 (82,92)

* *p* = 0.243 when comparing liver features with liver and spleen features; ** *p* = 0.278 when comparing liver features with liver and spleen features; *** *p* = 0.025 when comparing liver features with liver and spleen features; **** *p* = 0.024 when comparing liver features with liver and spleen features; ***** *p* < 0.001 when comparing liver features with liver and spleen features. Abbreviations: AUC = area under the receiver operating characteristic curve; CI = confidence interval.

**Table 3:** Top five weighted radiomic features of trained logistic regression classifiers. The feature names are based on the Pyradiomics library used for research [16].

Weight Ranking	Radiomic Feature Names							
	L1 Penalty and F0		L1 Penalty and F4		L2 Penalty and F0		L2 Penalty and F4	
	Feature Category	Feature Name	Feature Category	Feature Name	Feature Category	Feature Name	Feature Category	Feature Name
1st	GLRLM *	Low Gray Level Run Emphasis *	First order *	Robust Mean Absolute Deviation *	Shape *	Flatness *	NGTDM *	Coarseness *
2nd	Shape *	Flatness *	Shape ^#^	Maximum 2D Diameter Slice ^#^	Shape *	Least Axis Length *	Shape ^#^	Maximum 2D Diameter Slice ^#^
3rd	Shape *	Maximum 2D Diameter Row *	GLRLM *	Run Length Nonuniformity Normalized *	Shape *	Maximum 2D Diameter Row *	First order *	Robust Mean Absolute Deviation *
4th	GLSZ *	Zone Entropy *	NGTDM *	Coarseness *	GLRLM *	Low Gray Level Run Emphasis *	Shape ^#^	Surface Area ^#^
5th	Shape *	Least Axis Length *	GLCM ^#^	Cluster Shade ^#^	GLRLM *	Short Run Low Gray Level Emphasis *	Shape ^#^	Minor Axis Length ^#^

* liver radiomic features; ^#^ splenic radiomic features. Abbreviations: GLRLM = gray level run length matrix; NGTDM = neighboring gray tone difference matrix; GLSZM = gray level size zone matrix; GLCM = gray level co-occurrence matrix.

**Table 4:** Top five weighted radiomic features of trained tree-based ensemble classifiers. The feature names are based on the Pyradiomics library used for research [16].

Weight Ranking	Radiomic Feature Names					
	AdaBoosting		Gradient Boosting		XGBoosting	
	Feature Category	Feature Name	Feature Category	Feature Name	Feature Category	Feature Name
1st	First order *	Total Energy *	NGTDM *	Strength *	NGTDM *	Strength *
2nd	First order ^#^	Median ^#^	Shape *	Maximum 2D Diameter Row *	GLSZM *	Size Zone Nonuniformity *
3rd	GLRLM ^#^	Low Gray Level Run Emphasis ^#^	GLCM ^#^	Imc2 ^#^	GLRLM ^#^	Gray Level Variance ^#^
4th	GLSZM *	Size Zone Nonuniformity *	Shape *	Mesh Volume *	GLRLM *	Run Length Nonuniformity *
5th	Shape *	Flatness *	GLSZM *	Zone Variance *	GLSZM ^#^	Large Area High Gray Level Emphasis ^#^

* liver radiomic features; ^#^ splenic radiomic features. Abbreviations: NGTDM = neighboring gray tone difference matrix; GLSZM = gray level size zone matrix; GLRLM = gray level run length matrix; GLCM = gray level co-occurrence matrix.

## Data Availability

Additional data is available from the authors on request.

## Appendix A

### Appendix A.1. Machine Learning Classifier for Liver Fibrosis Staging

To avoid bias toward machine learning model selection, we chose a variety of commonly used classifiers for radiomics analysis, including multinomial logistic regression and tree-based ensemble classifiers.

To prevent overfitting on the training set, we used L1 (Lasso) and L2 (Ridge) regularization in the logistic regression classifier. Regularization can penalize high-valued regression coefficients and improves the generalizability of trained classifiers.

The tree-based ensemble classifier is a machine learning method that combines multiple decision trees to obtain the optimal classifier. In this study, classifiers were trained based on three typical ensemble methods, namely AdaBoosting [17], Gradient Boosting [18], and XGBoosting [19]. The classifiers were built by using Python 3.7.9 and library scikit-learn 0.23.2 [21].

### Appendix A.2. Radiomic Features Used by Trained Classifiers

To better understand the prediction made by machine learning classifiers, we evaluated the top 5 most important features of the trained classifiers.

As liver fibrosis has five stages ranging from F0 to F4, the multinomial logistic regression classifier consists of a set of formulae that calculate the probability (Pr) of each liver fibrosis stage:

$$\mathrm{Pr}\left(\mathrm{Fibrosis}\;\mathrm{stage}=0\right)=\frac{e^{\beta_{0}\cdot X_{i}}}{\sum_{i=0}^{4}e^{\beta_{i}\cdot X_{i}}}\;\;\;\;\;\;\;\;\;\;\mathrm{Pr}\left(\mathrm{Fibrosis}\;\mathrm{stage}=1\right)=\frac{e^{\beta_{1}\cdot X_{i}}}{\sum_{i=0}^{4}e^{\beta_{i}\cdot X_{i}}}$$

$$\mathrm{Pr}\left(\mathrm{Fibrosis}\;\mathrm{stage}=2\right)=\frac{e^{\beta_{2}\cdot X_{i}}}{\sum_{i=0}^{4}e^{\beta_{i}\cdot X_{i}}}\;\;\;\;\;\;\;\;\;\;\mathrm{Pr}\left(\mathrm{Fibrosis}\;\mathrm{stage}=3\right)=\frac{e^{\beta_{3}\cdot X_{i}}}{\sum_{i=0}^{4}e^{\beta_{i}\cdot X_{i}}}$$

$$\mathrm{Pr}\left(\mathrm{Fibrosis}\;\mathrm{stage}=4\right)=\frac{e^{\beta_{4}\cdot X_{i}}}{\sum_{i=0}^{4}e^{\beta_{i}\cdot X_{i}}}$$

Xi represents the radiomic features extracted from patient i, and β is the weight of radiomic features learned from the training set. When $\beta_{i}$ > 0, the corresponding feature is considered to be positively related to fibrosis stage i. As mentioned before, the top 5 most important features being positively related to fibrosis stages 0 and 4 were analyzed.

Gini importance is one of the common parameters when evaluating feature importance in tree-based ensemble classifiers [20]. The importance of each feature is computed as the total decrease in node impurity averaged over all decision trees in the ensemble classifier. We analyzed five features with highest Gini importance.
